# Case Report: Beyond conventional diagnostics: mNGS support in a complex immunocompromised patient diagnosis

**DOI:** 10.3389/fmed.2026.1791094

**Published:** 2026-05-05

**Authors:** Rossana Scutari, Valeria Fox, Martina Mastropaolo, Vanessa Fini, Marco Mussa, Paolo Bigliano, Luna Colagrossi, Gianluca Vrenna, Andrea Perinzano, Silvia Scabini, Carlo Federico Perno, Andrea Calcagno

**Affiliations:** 1 Multimodal Laboratory Research Unit, Bambino Gesù Children’s Hospital, IRCCS, Rome, Italy; 2 Microbiology and Diagnostic Immunology Unit, Bambino Gesù Children’s Hospital, IRCCS, Rome, Italy; 3 Unit of Infectious Diseases, Department of Medical Sciences, University of Turin, Turin, Italy; 4 SC Malattie Infettive, “Amedeo di Savoia” Hospital, ASL “Città di Torino”, Turin, Italy; 5 Unit of Infectious Diseases, AOU Città Della Salute e Della Scienza, Turin, Italy; 6 Department of Translational Medicine, University of Eastern Pidmont, Novara, Italy; 7 SCDO Malattie Infettive, AOU Maggiore Della Carità, Novara, Italy

**Keywords:** immunocompromised host, infectious diseases, microbiological diagnosis, mNGs, polymicrobial infection, T-cell lymphoma

## Abstract

Next-generation metagenomic sequencing (mNGS) enables the direct and unbiased detection of pathogens from clinical samples, overcoming the limitations of standard methods. It is particularly valuable in immunocompromised patients and in cases of complex infections. We report the case of a man in his 40s, born in North Africa, who was admitted with progressive skin and soft-tissue lesions after a minor foot trauma. The initially localized infection rapidly worsened, leading to bilateral pneumonia, acute respiratory failure, disseminated intravascular coagulation, and death. Histopathological examination revealed granulomatous inflammation with alcohol-resistant bacilli and an undiagnosed cutaneous T-cell lymphoma associated with hemophagocytic syndrome. Conventional microbiological tests identified multiple pathogens, including influenza A virus, herpes simplex virus 1 (HSV-1), *Candida albicans*, *Enterococcus faecalis*, *Proteus mirabilis*, and *Pseudomonas aeruginosa*; however, their heterogeneous distribution and isolation from non-sterile sites hindered etiological interpretation. Cultures and molecular assays for *Mycobacterium* species were negative despite findings of histological examination suggestive of granulomatous inflammation with alcohol-resistant bacilli. To clarify the diagnosis, mNGS was performed on blood, serum, and lymph node samples using host DNA depletion and Illumina sequencing. Bioinformatic analysis revealed a diverse microbial landscape, with the detection of *Fusarium pseudograminearum*, *Mycobacterium canettii,* and *Ralstonia* sp., alongside low-level viral sequences [Epstein–Barr virus (*EBV*) and *HSV-1*]. These results reflected the patient’s severe immune deficiency, characterized by a marked depletion of CD8^+^ T cells and NK cells. Although the results became available too late to influence treatment, mNGS provided crucial diagnostic insights, demonstrating its ability to uncover hidden or rare pathogens. Early application of mNGS could significantly improve diagnostic precision and therapeutic decisions in critically ill immunocompromised patients.

## 1 Introduction

Accurate and timely identification of pathogens is one of the most critical challenges in the management of infectious diseases, particularly in immunocompromised patients or those with atypical or polymicrobial infections. The complexity of these clinical pictures often stems from the overlap of different etiologies, previous exposure to antimicrobial treatments, and underlying immune dysfunction, factors that can hinder diagnostic interpretation and delay the initiation of appropriate therapy. In such contexts, infections can evolve rapidly and involve multiple anatomical sites, significantly increasing the risk of serious complications and mortality. Therefore, it is essential to have diagnostic tools that are fast, sensitive, and provide broad microbiological coverage (1, 2).

Conventional microbiological methods, including culture, targeted polymerase chain reaction (PCR), and serological tests, still form the basis of infectious disease diagnostics. Although cultures are essential for determining antimicrobial susceptibility, they are ineffective in cases involving non-viable microorganisms, microorganisms that are difficult to culture, or microorganisms that have already received antimicrobial treatments. Similarly, PCR and serological tests depend on preliminary diagnostic assumptions and are therefore limited to suspected pathogens, carrying the risk of failing to detect rare, emerging, or unexpected microorganisms. Furthermore, the sensitivity of these methods may be reduced in infections with a low microbial load or in cases where the infection is located deep within the body and the amount of microbial nucleic acid available is limited or degraded. These difficulties can lead to delays in diagnosis and frequent recourse to empirical therapies, which are often suboptimal (3–5).

In this scenario, next-generation metagenomic sequencing (mNGS) has emerged as a revolutionary diagnostic technology capable of directly identifying pathogens in clinical samples, eliminating the need for culture or prior diagnostic assumptions. Unlike targeted tests, mNGS uses a non-targeted sequencing approach, analyzing all nucleic acids (DNA and RNA) in the sample. This strategy allows microorganisms that are difficult to culture or undetectable by traditional methods to be identified and enables complex or polymicrobial infections to be characterized more quickly.

For critically ill or immunocompromised patients, for whom rapid diagnosis can guide life-saving interventions, this technology represents a paradigm shift toward precision infectious disease medicine.

However, despite its enormous potential, mNGS still has some critical issues. Environmental contamination, the presence of human DNA, and the detection of commensal microorganisms can complicate the interpretation of results and generate false positives. Therefore, an integrated analysis that considers the clinical and epidemiological features, carefully selects samples, and, when necessary, uses complementary confirmation methods is essential (6, 7).

An increasing amount of evidence shows that the early application of mNGS can significantly reduce the time needed to obtain an etiological diagnosis, enabling targeted and timely therapies and potentially improving prognosis (8, 9).

In this study, we present a clinical case in which mNGS played a pivotal role in identifying a complex infection that could not be diagnosed using conventional methods, highlighting the value of this emerging technology in clinical practice.

## 2 mNGS protocol

Inguinal lymph node, whole blood, and serum samples were collected from the patient according to standardized protocols for performing metagenomic next-generation sequencing (mNGS).

No chemical host DNA depletion was applied; instead, mechanical host depletion was performed before viral nucleic acid extraction. Specific extractions were then performed to isolate DNA from bacteria (both Gram-positive and Gram-negative species), fungi, and DNA viruses, as well as RNA from RNA viruses, to obtain a heterogeneous pool of microbial nucleic acids (Supplementary material).

Library preparation was carried out using a modified QIAseq FX Library Kit (Qiagen, Germany), optimized for low-input and clinically derived samples. Sequencing was subsequently performed on an Illumina NextSeq 550 platform to generate paired-end reads with a median depth of 269–732 million total reads per sample (732 M lymph node, 269 M blood, and 124 M serum), of which ~99.95% were human (Table 1).

**Table 1:** Distribution of sequencing reads across biological samples with human and microbial classification.

Material	Number of total reads	Number of human reads	Percentage of human reads	Number of non-human reads	Percentage of non-human reads	Number of bacterial reads	Percentage of bacterial reads (on non-human reads)	Number of viral reads	Percentage of viral reads (on non-human reads)	Number of fungal reads	Percentage of fungal reads (on non-human reads)
Lymph node	73,224,037	73,206,921	99.98	171,154	0,02	8,488	4.96	40	0.02	38,282	22.37
Blood	26,917,449	26,903,004	99.95	144,448	0,05	40,188	27.82	44	0.03	63,606	44.03
Serum	12,370,181	12,366,593	99.97	35,886	0,03	8,078	22.51	40	0.11	1,466	4.09

The resulting sequencing data were processed and analyzed using an in-house bioinformatic workflow (Supplementary material), which enabled high-resolution taxonomic classification. Additional quality control filters were applied to ensure robust and reliable interpretation of the results. Specifically, bacterial and fungal species identified by fewer than 50 reads or showing a relative abundance below 0.05% (with respect to total non-human reads) were excluded from the final dataset.

For eukaryotic viral species, no abundance or read-count filtering was applied, as viral nucleic acid loads in clinical samples are typically lower than those of bacteria or fungi. This approach also accounted for the potential clinical and epidemiological relevance of low-abundance viral species, particularly in immunocompromised hosts.

Finally, all taxonomically classified sequences were categorized according to pathogen type (i.e., bacteria, fungi, viruses, and parasites) and integrated with the patient’s clinical, histopathological, and microbiological data to support etiological interpretation.

## 3 Case presentation

We present the case of a man in his 40s, born in North Africa and living in Italy, who presented to the emergency department of a hospital in Turin (Italy). The starting complaint was swelling and redness of the left leg following a traumatic injury sustained 1 month earlier: the patient reported a laceration on the dorsal surface of his left foot while playing football in Italy. He had a recent trip to North Africa (Morocco) immediately after the injury. Initially presenting as localized erythema and swelling, the lesions progressively expanded to involve the entire ankle, leg, and thigh, evolving into diffuse dermo-hypodermitis. The course was slow but progressive, lasting approximately 7 months. The subdermal/skin lesions had worsened over time, with the development of nodules that opened, leaving necrotic subcutaneous scars, the largest (on the calf) being approximately 8 cm in diameter. Approximately 6 months after the initial presentation, the patient developed bilateral pulmonary involvement, evidenced by bibasal pulmonary lesions and a progressive decline in respiratory function, ultimately resulting in acute respiratory failure. The clinical course was further complicated by disseminated intravascular coagulation (DIC). Based on the lymphatic and pulmonary involvement, an initial working diagnosis of Epstein–Barr virus (EBV)-related lymphomatoid granulomatosis was considered; however, subsequent diagnostic investigations did not confirm this hypothesis.

Histopathological examination of four punch biopsies of the skin and soft tissues from the leg (including ulcerated and healthy tissues) revealed a chronic inflammatory process. The first biopsy demonstrated dermo-hypodermitis with granulomatous and lymphoplasmacytic infiltrates, along with acid-fast bacilli within immune cells, raising suspicion of a mycobacterial infection. At the following biopsies, obtained in the following months, a multinodular inflammatory infiltrate with aggregates of histiocytes and epithelial cells, surrounded by T lymphocytes and plasma cells, was observed. Multinucleated giant cells were also described, without evidence of caseous necrosis, consistent with granulomatous inflammation of likely infectious etiology. A large skin biopsy and histological analysis (performed 5 months after the initial presentation) revealed the presence of cutaneous T-cell lymphoma, complicated by hemophagocytic syndrome, a condition that explains the severe inflammatory activation and intravascular coagulation. B- and T-cell lymphomas were repeatedly searched in the initial biopsies and blood samples: no evidence of immune cells’ clonality was found; a profound CD8 + T-cell and natural killer cell deficit was observed and confirmed at further analysis, suggesting an unspecified immunodepression. Human immunodeficiency virus (HIV) infection was excluded by two negative serological tests performed during the observation period.

**Figure 1:**
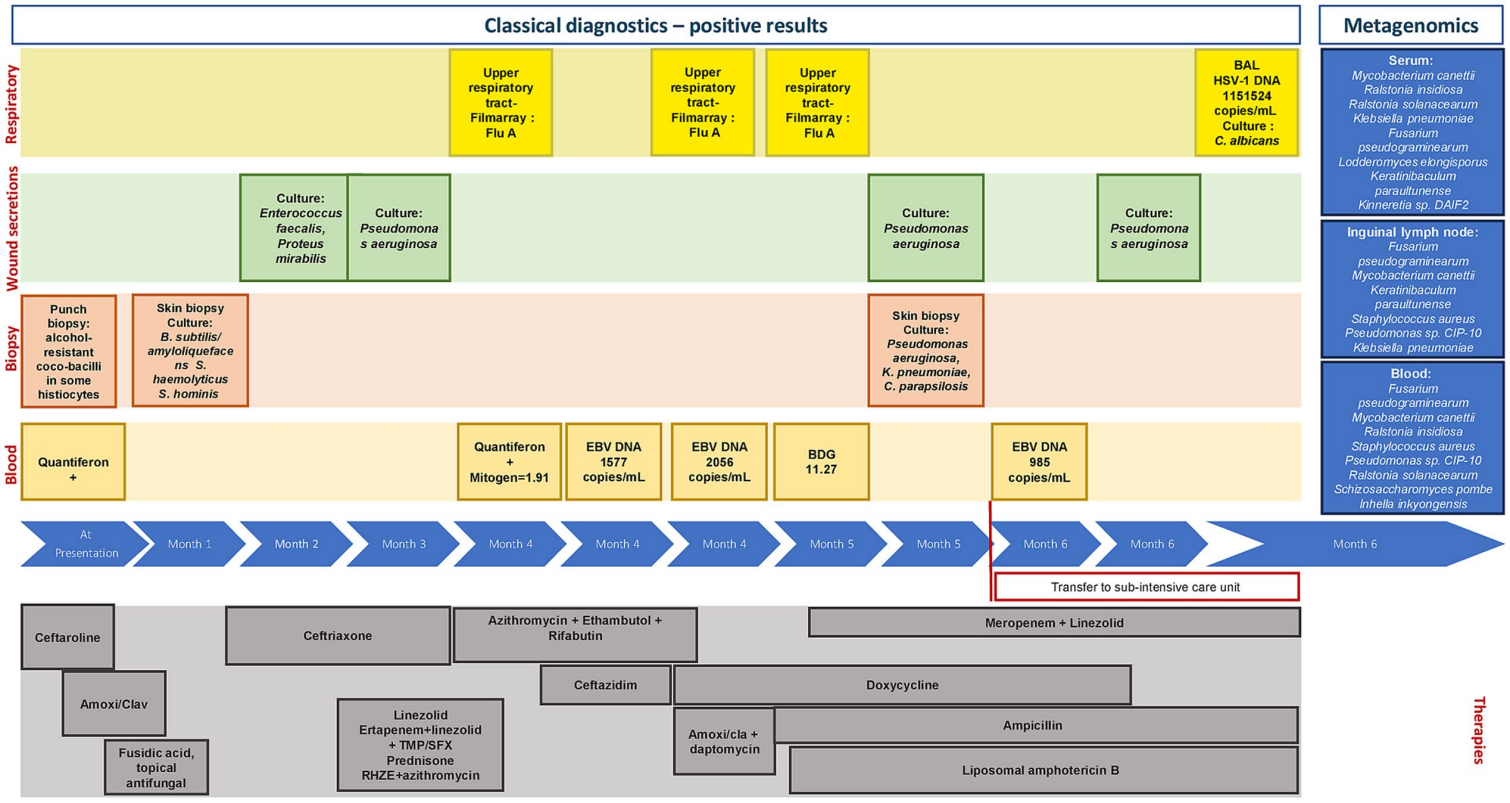
Timeline of microbiological positive results and patient treatment history during the observation period. BAL, Bronchoalveolar lavage; BDG, Beta-D-glucan; EBV, Epstein–Barr virus; Flu, Influenza virus; HSV, Herpes simplex virus.

Figure 1 also shows the multiple antibiotic and antifungal treatments that were administered. These treatments included both empirical and pseudo-targeted therapies that were prescribed considering the skin and foot tissue infection, with the potential involvement of Actinomycetes, isolated Gram-negative bacteria, and fungal agents. First-line anti-mycobacterial agents and low-dose prednisolone were also administered for approximately 4 weeks, with fever improvement but no change in the skin lesions.

The initially localized lesion slowly progressed, causing significant damage to the skin and soft tissues, eventually leading to systemic manifestations of an infection, including bilateral pulmonary involvement, acute respiratory failure, disseminated intravascular coagulation, and ultimately death (approximately 7 months after the initial presentation).

## 4 Microbiological investigation and relevant findings

Before performing mNGS, the patient underwent extensive conventional microbiological testing. Several pathogens were detected, but their marked heterogeneity did not point to a single predominant infection or clearly explain the patient’s clinical course. This unusually broad and dispersed pattern itself suggested a state of profound immunosuppression rather than a single causative agent.

As summarized in Figure 1, some findings nonetheless provided partial diagnostic clues. At the respiratory level, influenza A infection was initially documented, followed by a high HSV-1 DNA load and *Candida albicans* growth in bronchoalveolar lavage (BAL) fluid. Pus cultures from cutaneous lesions first revealed *Enterococcus faecalis* and *Proteus mirabilis*, while *Pseudomonas aeruginosa* was subsequently isolated from multiple samples, including skin biopsies. In some instances, this organism was detected in association with *Klebsiella pneumoniae* and *Candida parapsilosis*. The presence of multiple opportunistic pathogens across non-sterile body sites, without evidence of a dominant invasive agent, supported the interpretation of secondary colonization in the context of severe immune dysfunction.

Blood investigations demonstrated a positive Quantiferon-TB Gold test with reduced mitogenic response, suggesting previous exposure to *Mycobacterium tuberculosis* complex bacteria and a reduced immune response; low-level Epstein–Barr virus (EBV) viremia was also observed.

Histopathological examination of skin punch biopsies revealed acid-fast bacilli within immune cells, raising suspicion for a mycobacterial infection, although both culture and targeted molecular assays were negative for *Mycobacterium tuberculosis* complex and non-tuberculous mycobacteria.

Histopathological examination reported skin sections with intact epidermis covering extensively necrotic with dermal connective tissue and subcutaneous adipose tissue with abundant nuclear debris. In some areas, atypical lymphoid cells were observed, heterogeneous in shape and distribution. The majority of the small and medium-sized cells and the vast majority of the large, more irregular cells are cluster of differentiation 3 (CD3)-positive T cells; they also diffusely coexpress granzyme, showed only partial expression of CD5, CD2, CD7, and T-cell intracellular antigen-1 (TIA1), showed weak and partial expression of CD4 and programmed cell death protein 1 (PD1). CD30 staining is positive in a significant portion of medium and large lymphoid cells, mostly arranged around vessels. Staining for CD79 alpha revealed the presence of a component of small-, medium-, and large-sized positive B cells arranged in a hazy nodule within a necrotic setting, and a large proportion of them tested positive for EBV viral RNA using the Epstein–Barr virus-encoded RNA probe with Chromogenic *in situ* hybridization. The pathologist was unable to distinguish between a form of EBV-positive B-cell lymphoma with pleomorphic reactive T cells, such as lymphomatoid granulomatosis, supported by the presence of a clonal rearrangement of the immunoglobulin gene, detected by molecular testing, and a form of angiocentric T-cell lymphoma with a partial cytotoxic phenotype, supported by the loss of T-cell markers.

The complexity of the results, characterized by a heterogeneous mix of pathogens, including organisms that may act as bystanders in non-sterile body sites, did not converge into a single explanatory etiology. Moreover, the only partial response to targeted therapies based on these isolations further suggested that none of them represented the primary driver of disease. This uncertainty prompted further investigation with mNGS. Therefore, samples for mNGS were collected 1 week before his transfer to the intensive care unit (ICU).

## 5 Metagenomic analysis

Metagenomic analysis on lymph node, blood, and serum samples revealed distinct microbial profiles across the three compartments, highlighting the presence of both opportunistic and environmental microorganisms (Table 2). Among the most abundant organisms detected, *Fusarium pseudograminearum*, a fungal species that dominated non-human reads in both lymph node and blood samples, was also detected in serum, albeit at lower abundance. This suggests systemic dissemination or a high fungal load, particularly in the bloodstream. *Fusarium pseudograminearum* is a filamentous fungus typically associated with soil and cereal crops; its detection in the analyzed samples suggests a likely environmental origin, plausibly introduced through the traumatic event. This genus is known to cause infections predominantly in immunocompromised patients and those with hematological malignancies, in whom the portal of entry often involves tissue disruption or mucosal barrier breakdown (10). This infection may have helped create conditions that facilitated the entry and proliferation of other opportunistic microorganisms.

**Table 2:** Species-level taxonomic classification of non-human reads across samples.

Species	Metric	Lymph node	Blood	Serum
*Fusarium pseudograminearum*	n reads	28,014	59,694	248
	Relative abundance on non-human reads (%)	16.37	41.33	0.69
	K-mer counts	97,857	98,588	33,621
*Mycobacterium canettii*	n reads	106	82	18
	Relative abundance on non-human reads (%)	0.06	0.06	0.05
	K-mer counts	22,022	13,774	4,200
*Ralstonia* sp.	n reads	nd	1724	888
	Relative abundance on non-human reads (%)	nd	1.19	2.47
	K-mer counts	nd	30,165	15,696
*Lodderomyces elongisporus*	n reads	544	292	226
	Relative abundance on non-human reads (%)	0.32	0.20	0.63
	K-mer counts	1,412	675	304
*Schizosaccharomyces pombe*	n reads	nd	650	28
	Relative abundance on non-human reads (%)	nd	0.45	0.08
	K-mer counts	nd	686	33
*Inhella inkyongensis*	n reads	nd	130	nd
	Relative abundance on non-human reads (%)	nd	0.09	nd
	K-mer counts	nd	1,347	nd
*Human alphaherpesvirus 1*	n reads	nd	14	16
	Relative abundance on non-human reads (%)	nd	0.01	0.04
	K-mer counts	nd	236	354
*Human gammaherpesvirus 4*	n reads	36	12	6
	Relative abundance on non-human reads (%)	0.02	0.01	0.02
	K-mer counts	931	354	117

K-mer counts, number of distinct k-mers associated with each taxon as reported from kraken2; nd, not detected.

*Mycobacterium canettii*, a member of the *Mycobacterium tuberculosis* complex, was consistently identified across all sample types, with the highest relative abundance in serum.

Additional bacterial species included *Ralstonia* sp., predominantly found in blood and serum, and *Inhella inkyongensis*, sporadically detected in blood samples. Rare fungal species, such as *Lodderomyces elongisporus* and *Schizosaccharomyces pombe*, were sporadically detected, primarily in serum and blood samples.

Human herpesviruses, including Epstein–Barr virus (*Human gammaherpesvirus* 4) and *Human alphaherpesvirus* 1, were also identified, albeit at very low levels.

All microbial species identified across the different compartments are detailed in Table 2.

Overall, the metagenomic data reveal a diverse microbial landscape, predominantly composed of fungal and bacterial reads, with a relatively minor viral component. These findings underscore a complex scenario, with the detection of pathogenic bacteria, opportunistic species, and fungi, some of which had not been identified by conventional diagnostic tests. This highlights the added diagnostic value of mNGS, particularly in capturing a broader infectious spectrum and offering improved diagnostic insight, particularly in immunocompromised hosts.

## 6 Discussion and conclusion

The clinical case presented highlights the diagnostic value of mNGS in an immunocompromised patient with a complex and evolving infection that could not be clarified by conventional diagnostic approaches. Despite extensive microbiological and histopathological investigations, the markedly heterogeneous mixture of pathogens, many of which were associated with only a partial response to targeted therapies, did not point to a single coherent etiology. The use of mNGS enabled the simultaneous detection of bacterial, fungal, and viral pathogens, including organisms not identified by traditional techniques (6, 11), thereby providing a more comprehensive understanding of the infectious landscape.

The identification of *Mycobacterium canettii*, a rare member of the *Mycobacterium tuberculosis complex* (MTBC), is of particular clinical and microbiological relevance. This species, known for its geographical distribution apparently limited mostly (though not only; see below) to the Horn of Africa, has been described primarily in immunocompetent patients with localized forms of disease, such as lymphadenitis or skin lesions (12). However, cases of disseminated infections have also been reported, particularly in immunocompromised individuals (13, 14). Its identification, despite negative results from cultures and specific molecular tests, is consistent with histological findings (presence of acid-fast bacilli) and the positive Quantiferon test; it highlights the ability of mNGS to detect causative agents that are otherwise “invisible” to traditional tools, as also demonstrated by similar cases described in the literature (15). This finding is epidemiologically consistent with the patient’s recent travel to Morocco in the summer, a region where *Mycobacterium canettii* exposure, although rare, has been documented, thereby reinforcing the clinical relevance of this diagnosis (16).

At the same time, the predominant detection of *Fusarium pseudograminearum* in lymph nodes and blood suggests active fungemia or deep colonization, consistent with the clinical picture of necrotizing dermo-hypodermitis and immunosuppression. Although *F. pseudograminearum* is traditionally known as a phytopathogen responsible for crown rot in cereals, its presence in human clinical samples is extremely rare and is not widely documented in the medical literature (17).

However, the *Fusarium* genus is well recognized for its ability to cause serious invasive infections in immunocompromised patients, with higher mortality rates in disseminated forms (18, 19). *Fusarium* spp. infections often manifest with persistent fever, metastatic skin lesions, and pulmonary involvement, particularly in individuals with prolonged neutropenia or severe cellular immunodeficiency. In these patients, fungemia is a common manifestation and can be confirmed by positive blood cultures, although diagnosis is often hampered by the difficulty of isolation and similarity to other filamentous fungi (20).

The detection of *F. pseudograminearum* in three biological compartments (lymph node, blood, and serum), with particular abundance in blood, raises questions about its pathogenic potential in conditions of extreme immunosuppression. Although there are no confirmed clinical cases of human infection with this specific species, its presence may have changed the antifungal prescription (liposomal amphotericin B was prescribed quite late, when the pulmonary and systemic damage was already severe). Consistent with our findings, the patient also showed a positive beta-D-glucan result (Figure 1) despite the absence of fungal growth in conventional cultures.

The detection of other microorganisms, including *Ralstonia* sp., reflects the complexity of the pathogenic microbiome in immunocompromised patients. These agents are known for their involvement in nosocomial and opportunistic infections (21–23), and their co-presence may have contributed to clinical deterioration, although their direct pathogenic role cannot be established with certainty.

Notably, several of these microorganisms detected, including *F. pseudograminearum* and *Ralstonia* spp., are commonly found in soil and other environmental sources, suggesting how the patient’s skin lesion exposure to soil may have facilitated the entry of these pathogens, probably also aided by the context of profound immunosuppression.

In addition, low-load human viruses such as Epstein–Barr virus (EBV) and herpes simplex virus 1 (HSV-1), probably reactivated, were identified.

Interpretation of diverse microbial profiles in low-biomass samples remains a known diagnostic challenge, necessitating the rigorous exclusion of background contaminants. To minimize this impact, a multifaceted validation approach was adopted: beyond bioinformatic filtering, the pathogenic relevance of our findings is corroborated by consistent results across independent sterile sites. In this case, the alignment between mNGS data and the patient’s clinical, biochemical (e.g., beta-D-glucan), and histopathological features effectively mitigates the risk of environmental noise, supporting a true polymicrobial infection.

The abundance and diversity of microorganisms detected by mNGS are difficult to reconcile with a state of immunocompetence. In this case, the presence of systemic T-cell lymphoma associated with hemophagocytic syndrome clarifies the pathogenetic basis of the complex infectious picture found. The lymphoma presumably caused severe impairment of cellular immunity, favoring the proliferation and dissemination of multiple bacterial, fungal, and viral species. This scenario highlights how immune dysregulation can profoundly alter host–pathogen dynamics, transforming otherwise marginal infections into disseminated and polymicrobial processes. In this patient, the coexistence of lymphoma and widespread microbial dissemination illustrates the close association between immune deficiency and infectious complexity, underscoring the importance of interpreting mNGS results within an integrated clinical and immunological context.

Our findings are consistent with previous case studies in which metagenomic sequencing identified unexpected pathogens that were not detectable by conventional tests. In the case of secondary hemophagocytic syndrome associated with visceral leishmaniasis described by Chen et al. (24), mNGS was instrumental in identifying the causative agent and preventing a potentially fatal outcome. Similarly, Wei et al. (25) described a complex polymicrobial infection in a liver transplant recipient in which mNGS revealed the presence of rare pathogens (*Cunninghamella elegans*, *Bordetella bronchiseptica*, and *Pneumocystis jirovecii*) not detected by traditional diagnostic methods, confirming the value of this approach in critically ill or immunocompromised patients. Finally, Duan et al. (26) reported a case of severe pulmonary infection in a non-HIV immunocompromised patient, where mNGS identified a complex polymicrobial etiology that had remained undetected through conventional microbiological testing, underscoring the value of mNGS in revealing hidden co-infections.

Moreover, recent studies have demonstrated that early implementation of mNGS significantly enhances diagnostic performance compared to conventional microbiological methods (27–29). A study involving septic patients revealed that early mNGS application was associated with a reduced risk of early mortality (within 7 days), although it did not significantly impact 28-day survival rates, underscoring the importance of timely deployment in critical care settings (30). In the context of pediatric oncology patients with suspected bloodstream infections, mNGS detected pathogens in 74.3% of cases and 20% by blood cultures, including fungi missed by conventional methods, directly impacting treatment decisions (31).

In our case, although mNGS provided a clear interpretative key and documented the presence of a disseminated polymicrobial infection, the analysis was requested too late, when the clinical condition was already compromised, ultimately leading to his death. Consequently, the delayed turnaround time limited the potential for targeted therapeutic intervention. This highlights not only the high sensitivity and breadth of the diagnostic spectrum of mNGS but also the fundamental importance of its early integration into diagnostic pathways, particularly in immunocompromised patients or those with rapidly evolving infections.

Our study has some limitations. First, the use of k-mer-based classifiers for the taxonomic classification of reads may yield imprecise species-level assignments, particularly for fungal taxa, due to database biases and close phylogenetic relationships. The detection of *Fusarium pseudograminearum*, a species not previously linked to human infections, has therefore been retained but should be interpreted cautiously, with a more conservative interpretation focusing on the genus level (*Fusarium* spp.). Additionally, the detection of *Ralstonia* sp. should also be interpreted cautiously due to their frequent occurrence as environmental contaminants or reagent impurities in metagenomic sequencing kits and laboratory settings. However, given its high relative abundance and absence in negative controls, we retained it at the genus level as clinically plausible in this immunocompromised patient.

In conclusion, this case demonstrates the effectiveness of mNGS in diagnosing complex infections in immunocompromised patients due to its ability to identify a wide spectrum of pathogens, including those undetectable by conventional methods. The results obtained may have clarified the etiology of the clinical picture and guided treatment, unfortunately, at a too advanced stage of the disease. The experience, therefore, highlights not only the diagnostic value of mNGS but also the critical importance of its timely application to improve prognosis in complex clinical settings.

## Data Availability

The data presented in the study are deposited in the European Nucleotide Archive (ENA) repository, accession number PRJEB106795.
