# Supplementary material for: Case Report: Beyond conventional diagnostics: mNGS support in a complex immunocompromised patient diagnosis
Source: Front Med (Lausanne). 2026 May 5;13:1791094. doi: 10.3389/fmed.2026.1791094 (PMC13183559; doi:10.3389/fmed.2026.1791094)
Supplement: Supplementary file 1 [file Data_Sheet_1.docx]

**Supplementary methods**

**Sample preparation and nucleic acid extraction**

To identify the heterogeneous microbial population (comprising viruses, bacteria and fungi) present in the the different clinical samples, nucleic acids were extracted using three distinct protocols optimized for viral, bacterial, and fungal targets

*Viral DNA/RNA extraction*

Viral genome isolation was preceded by an enrichment step aimed at concentrating virus-like particles (VLPs). This was achieved using a homemade pre-extraction protocol based on centrifugation followed by filtration through a 0.45 µm polyethersulfone filter, which mechanically removes most human and bacterial cells prior to nucleic acid extraction. The resulting filtrate was then used for nucleic acid extraction with the QIAamp MinElute Virus Spin Kit (Qiagen, Hilden, Germany) according to the manufacturer's instructions.

*Bacterial DNA extraction*

Bacterial genome isolation was performed directly from clinical specimens using a “home made” pre-extraction protocol to facilitate cell wall lysis of Gram-positives and Gram-negatives by enzymatic digestion with lysozyme. Bacterial DNA extraction was subsequently performed using the EZ2 automated extractor (Qiagen BioRobot EZ2) with the appropriate extraction kit (EZ1&2 DNA Tissue kit, Qiagen) following the manufacturer's instructions. No chemical host DNA depletion step was applied during this extraction.

*Fungal DNA extraction*

The fungal genome was isolated directly from clinical specimens using a homemade pre-extraction protocol capable of lysing fungal cell walls by enzymatic digestion. Fungal DNA was then extracted using an EZ2 automated extractor (Qiagen BioRobot EZ2) and the appropriate extraction kit (EZ1&2 DNA Tissue Kit, Qiagen), following the manufacturer's instructions. Consistent with the bacterial workflow, no specific host DNA depletion step was applied.

**Amplification and sequencing**

Metagenomic analysis was performed on all samples collected using a modified QIAseq FX Library Kit (Qiagen, Germany) and sequenced through Illumina technology on a NextSeq 500 platform.

In particular, this protocol included 3 main steps: i) Amplification of total viral RNA and DNA from purified nucleic acid. This step involved denaturation of RNA, reverse transcription, and amplification of the whole RNA transcriptome with final cDNA production; ii) Enzymatic fragmentation and library generation. The cDNA obtained from the previous step was first subjected to enzymatic fragmentation and then used for library preparation using the QIAseq FX Single Cell RNA Library Kit (Qiagen); iii) Illumina sequencing. The pool was loaded at a concentration of 1.8 pM onto an Illumina High Output Flow v.2.5 cell cartridge. Samples were then sequenced with the NextSeq 550 instrument (Illumina, San Diego, CA, USA) with 2 × 150-bp paired-end reads.

To exclude possible contamination, negative controls were added.

**Bioinformatic analysis**

Demultiplexed raw reads were trimmed for adapter and quality (Phred score > 28) and deduplicated using Fastp (v0.23.2) [1]. The final reads quality was assessed by FastQC (v0.11.9) and MultiQC (v1.12) [2-3]. Reads mapping to the human genome (GRCh38) were removed using bowtie2 (v2.4.4) [4]. Taxonomy was assigned using Kraken 2 (v2.1.2) [4] with confidence score set to 0.1 and flag “ --report-minimizer-data" to estimate the number of distinct k-mers associated with each taxon, using two databases: i) Standard-16, including mainly bacteria and viruses, and ii) standard for fungi, and interactively visualized by pavian (v1.0) [5]. Species observed in the blanks were removed from all samples. Species observed in the procedural blanks (processed alongside each extraction batch) were removed from all samples using a conservative approach to eliminate potential reagent- or laboratory-derived contaminants. Specifically, any taxon detected in the negative controls was entirely excluded from the corresponding clinical sample analysis, regardless of its read count, to maximize specificity. An additional filtering was performed by discarding bacterial and fungal species that were identified by less than 50 reads and with a relative abundance (related to the total number of non-human reads) of less than 0.05%. For eukaryotic viral species, no filtering thresholds were applied regarding read counts or relative abundance, since viruses typically have lower loads in clinical samples compared to bacteria and fungi, and due to the potential clinical and epidemiological relevance of low-abundance viral species.

**References**

1. Chen S, Zhou Y, Chen Y, Gu J. fastp: an ultra-fast all-in-one FASTQ preprocessor. Bioinformatics. 2018 Sep 1;34(17):i884-i890. doi: 10.1093/bioinformatics/bty560
2. Andrews, Simon. "FastQC: a quality control tool for high throughput sequence data." (2010).
3. Ewels P, Magnusson M, Lundin S, Käller M. MultiQC: summarize analysis results for multiple tools and samples in a single report. Bioinformatics. 2016 Oct 1;32(19):3047-8. doi: 10.1093/bioinformatics/btw354.
4. Langmead, B., Salzberg, S. Fast gapped-read alignment with Bowtie 2. Nat Methods 9, 357–359 (2012). https://doi.org/10.1038/nmeth.1923
5. Florian P Breitwieser, Steven L Salzberg, Pavian: interactive analysis of metagenomics data for microbiome studies and pathogen identification, Bioinformatics, Volume 36, Issue 4, February 2020, Pages 1303–1304, https://doi.org/10.1093/bioinformatics/btz715
